# Identification of Forensically Important Calliphoridae and Sarcophagidae Species Collected in Korea Using SNaPshot Multiplex System Targeting the Cytochrome c Oxidase Subunit I Gene

**DOI:** 10.1155/2018/2953892

**Published:** 2018-02-28

**Authors:** Ji Hye Park, Sang Eon Shin, Kwang Soo Ko, Seong Hwan Park

**Affiliations:** ^1^National Forensic Service, Wonju, Republic of Korea; ^2^Department of Legal Medicine, Korea University College of Medicine, Seoul, Republic of Korea

## Abstract

Estimation of postmortem interval (PMI) is paramount in modern forensic investigation. After the disappearance of the early postmortem phenomena conventionally used to estimate PMI, entomologic evidence provides important indicators for PMI estimation. The age of the oldest fly larvae or pupae can be estimated to pinpoint the time of oviposition, which is considered the minimum PMI (PMI_min_). The development rate of insects is usually temperature dependent and species specific. Therefore, species identification is mandatory for PMI_min_ estimation using entomological evidence. The classical morphological identification method cannot be applied when specimens are damaged or have not yet matured. To overcome this limitation, some investigators employ molecular identification using mitochondrial cytochrome c oxidase subunit I* (COI)* nucleotide sequences. The molecular identification method commonly uses Sanger's nucleotide sequencing and molecular phylogeny, which are complex and time consuming and constitute another obstacle for forensic investigators. In this study, instead of using conventional Sanger's nucleotide sequencing, single-nucleotide polymorphisms (SNPs) in the* COI* gene region, which are unique between fly species, were selected and targeted for single-base extension (SBE) technology. These SNPs were genotyped using a SNaPshot® kit. Eleven Calliphoridae and seven Sarcophagidae species were covered. To validate this genotyping, fly DNA samples (103 adults, 84 larvae, and 4 pupae) previously confirmed by DNA barcoding were used. This method worked quickly with minimal DNA, providing a potential alternative to conventional DNA barcoding. Consisting of only a few simple electropherogram peaks, the results were more straightforward compared with those of the conventional DNA barcoding produced by Sanger's nucleotide sequencing.

## 1. Introduction

Estimation of postmortem interval (PMI) is important in unusual death cases. Various methods relying on early postmortem changes, such as livor mortis, rigor mortis, and body cooling, have been used to estimate PMI [1]. Estimation of PMI using insects is important for late postmortem changes. Medicolegal entomology focuses primarily on providing evidence of the amount of time during which a corpse or carcass has been exposed to colonization by insects, which helps to estimate the minimum postmortem interval (PMI_min_) [2, 3]. The first arrivers at a carcass are usually flies (order Diptera), especially blowflies (family Calliphoridae) [4].

In general, forensically important fly families include Calliphoridae, Sarcophagidae, Muscidae, and Piophilidae [5]. The family Calliphoridae is the taxon of greatest significance in forensic entomology. According to the first survey of forensically important entomofauna collected from medicolegal autopsies in South Korea, the predominant family of necrophagous flies was Calliphoridae and the second Sarcophagidae [6]. We selected 11 Calliphoridae and 7 Sarcophagidae species mainly based on the list from a previous study in South Korea [7]. One Sarcophagidae species,* S. crassipalpis*, was added based on the literature [6, 8].

A morphology-based identification method has traditionally been used to identify forensically important fly species. However, morphology-based identification has limitations. First, the fly obtained from the crime scene may lack the characteristics necessary for identification because of damage. Second, the taxonomic literature regarding immature stage samples is currently insufficient. Third, rearing samples to adult stages is time consuming. Last, identification of closely related sister species can cause confusion [9]. Accordingly, molecular identification methods utilizing nucleotide sequence comparison have been proposed as alternatives. DNA-based methods for species identification can solve these problems, especially for scientists who are not formally trained in taxonomy, and can be applied to all life stages and sample types, including ancient or damaged samples whose morphological characteristics have been destroyed [10, 11].

The molecular identification of fly species using a variety of gene regions has been researched [12–14]. The mitochondrial cytochrome c oxidase subunit I* (COI)* gene region has been the region most commonly used for insect identification due to its high degree of interspecies nucleotide variation [15–17]. Moreover, the properties of the mitochondrial* COI* gene are maternally inherited with no recombination event, and these gene regions are easy to amplify because of their high copy numbers. Unlike nuclear genes, these genes lack noncoding regions and are highly conserved among phyla [18]. Therefore, we have chosen single-nucleotide polymorphisms (SNPs) within the* COI* gene region that can discriminate between species of flies.

Conventionally, the molecular identification method has used Sanger's nucleotide sequencing to identify forensically important fly species. This method involves a complicated and time-consuming process. Therefore, a variety of other molecular techniques for identification have been reported, such as RFLP (restriction fragment length polymorphism) and AFLP (amplified fragment length polymorphism) [12, 13]. However, identification based on these techniques relies on a complicated decoding process, and throughput is too low [19]. Because many forensic samples at crime scenes exist in small amounts or in degraded condition, a new method that does not require Sanger's sequencing would be beneficial [20–22].

We used the single-base extension (SBE) method with fluorescence intensity detection (SNaPshot multiplex system), which is one of the SNP genotyping methods. The SBE method with fluorescence intensity detection has the advantages of a high success rate, the capacity for multiplex, a reasonable price, and universal application [23]. To our knowledge, this is the first adoption of SBE technology for identification of forensically important flies.

## 2. Materials and Methods

### 2.1. Sample Collection

Ninety-seven adult flies, 84 larvae, and 4 pupae were collected from Jeju Island, Jeollanam-do Province, Gyeonggi-do Province, and Seoul, South Korea. One hundred sixteen Calliphoridae flies were collected, and 69 Sarcophagidae flies were collected. The Calliphoridae species were* Lucilia ampullacea *(Villeneuve),* Lucilia caesar *(Linnaeus),* Lucilia illustris* (Meigen),* Calliphora lata *(Coquillett),* Calliphora vicina *(Robineau-Desvoidy),* Chrysomya megacephala *(Fabricius),* Chrysomya pinguis *(Walker),* Lucilia sericata *(Meigen),* Phormia regina *(Meigen),* Aldrichina grahami *(Aldrich), and* Triceratopyga calliphoroides *(Rohdendorf). The Sarcophagidae species were* Parasarcophaga albiceps* (Meigen),* Sarcophaga similis* (Meade),* Sarcophaga haemorrhoidalis* (Fallén),* Sarcophaga peregrina* (Robineau-Desvoidy),* Sarcophaga melanura* (Meigen),* Sarcophaga dux* (Thomson), and* Sarcophaga crassipalpis* (Macquart). Adult samples of each species were identified morphologically. The species of larvae and pupae were identified using molecular barcoding targeting the* COI* gene region.

### 2.2. DNA Extraction

DNA was extracted using a GeneAll Tissue SV Mini Kit (GeneAll, Seoul, Korea). The method followed the manufacture's protocols in the kit for relevant sample types. A nondestructive DNA extraction method was used for adult fly samples to preserve voucher specimens [24]. The samples of larva and pupa were destroyed and exhausted for DNA extraction.

### 2.3. Selection of Species-Specific SNPs

To select fly species-specific SNPs, full-length nucleotide sequences of the* COI* gene from 18 fly species (11 species of Calliphoridae, 7 species of Sarcophagidae) were collected from the NCBI GenBank (http://www.ncbi.nlm.nih.gov/nuccore/). Additionally, the fly samples were searched with the Basic Local Alignment Search Tool (BLAST) at the National Center for Biotechnology Information. To exclude intraspecific SNPs from the targeted interspecific SNPs, the sequences of each species were aligned using MEGA 5.10 software, and a representative consensus sequence of each species was generated. The accession numbers retrieved from the GenBank data are shown in Table 1. The International Union of Pare and Applied Chemistry (IUPAC) nucleic acid code was used to indicate nucleotide degeneracy in the consensus sequences. The Calliphoridae and Sarcophagidae samples (97 adults, 84 larvae, and 4 pupae) were analyzed using Sanger's nucleotide sequencing with previously announced study primer sets [6]. The consensus sequences were created by alignment, and then SNPs were selected based on interspecies variation. Following the consensus sequence, 6 SNP markers that can distinguish Calliphoridae 11 species were selected.

### 2.4. Species Identification with the SNaPshot Multiplex System

#### 2.4.1. SNaPshot Template Amplification by Singleplex PCR according to Family

To amplify the mitochondrial* COI* locus, which contains the fly species-specific SNPs, two primer pairs were designed. One was for the Calliphoridae, and the other was for the Sarcophagidae. The Calliphoridae species primer pair (CA-SNP) was designed for the front section of the* COI* gene. The other primer pair for the Sarcophagidae species (SA-SNP) was targeted to the end of the* COI* gene sequence. When the secondary structure and extent of self-complementarity were identifiable, the primer pairs were confirmed using Primer3 (http://bioinfo.ut.ee/primer3/). The sequences of the two primer pairs from 5′ to 3′ are shown in Table 2. Amplifications of genes from each family were performed in a total volume of 20 *μ*L, containing Gold ST★R 10x Buffer (Promega, Madison, WI, USA), 5 units of AmpliTaq Gold® DNA polymerase (Promega), 0.8 *μ*M of CA-SNP or SA-SNP primer set, and sterile water. Polymerase chain reaction (PCR) amplifications were conducted in a 2720 Applied Biosystems thermal cycler (Foster City, CA, USA). The conditions of the thermal cycler were as follows: initial denaturation at 95°C for 11 min, 33 cycles of denaturation at 94°C for 20 sec, annealing at 50°C for 1 min, extension at 72°C for 30 sec, and a final extension at 72°C for 7 min. The PCR products were detected by gel electrophoresis in a 2% agarose gel to ensure the expected size and fragment quality. The remaining PCR products were purified to remove excess PCR primers and dNTPs using ExoSAP-IT reagent (Affymetrix, Santa Clara, CA, USA), which effectively degrades PCR primers and dNTPs, following the manufacturer's protocol.

#### 2.4.2. SNaPshot Multiplex Reaction

SBE multiplex primers targeting interspecific SNPs were designed for each family, that is, Calliphoridae and Sarcophagidae. The set for the Calliphoridae species was composed of 6 SBE primers designed to bind contiguously to the SNPs in the forward direction, and the set for the Sarcophagidae family consisted of 4 SBE primers designed to bind neighboring SNPs in the reverse direction. In the case of Calliphoridae, a few different versions of SBE primers targeting the same sites were designed because of interspecific variation between species. The list of primers is shown in Table 3. The possibility of secondary structure and self-complementarity of the primers was checked using Primer3 (http://bioinfo.ut.ee/primer3/). The various primer sizes were obtained by adding poly T-tails of different lengths at the 5′ end of the primers, from 25 to 75 bp in Calliphoridae and 26 to 56 bp in Sarcophagidae. These methods are designed for Calliphoridae and Sarcophagidae samples of which families are morphologically identified. Therefore, if the family of a sample is unknown, these methods are not applicable. Using the SNaPshot multiplex kit (Applied Biosystems), the multiplex reactions were performed in a 10-*μ*L solution containing 3 *μ*L SNaPshot Multiplex Ready Reaction mix of fluorescent dideoxynucleotides (Green; A = dR6G, Black; C = dTAMRA™, Blue; G = dR110, Red; T = dROX™), 1 *μ*L PCR template, 5 *μ*L sterile water, and 1 *μ*L extension primer mix. The respective primer concentrations in the multiplex reaction are shown in Table 3. The SNaPshot reactions were performed in a 2720 thermal cycler (Applied Biosystems). The conditions of the thermal cycler were as follows: repeat for 25 cycles of 96°C for 10 sec, 55°C for 5 sec, and 60°C for 30 sec. The products were held at 4°C until postextension treatment. To remove residual ddNTPs and primers, SNaPshot products were purified with Alkaline Phosphatase, Calf Intestinal (CIP) by adding 1 unit of CIP into the SNaPshot reaction. The mixture was incubated at 37°C for 60 min, and then the CIP was deactivated by incubation at 80°C for 15 min.

#### 2.4.3. Capillary Electrophoresis and Product Analysis

The purified SNaPshot products were mixed with 9.4 *μ*L of formamide and 0.1 *μ*L of GeneScan-120 LIZ size standard (Applied Biosystems). The products were denatured by keeping them at 95°C for 5 min and were then placed on ice or at 4°C until loading. Electrophoresis on the ABI PRISM 3500 Genetic Analyzer was set up with a 36-cm capillary array and POP-4 polymer to load SNaPshot multiplex reaction products. All results were analyzed using GeneMapper software v5.0.

## 3. Results

### 3.1. Selection of Fly Species-Specific SNPs

Complete mitochondrial* COI* gene sequences from 18 fly species were collected from GenBank (Table 1). Based on the sequences, 6 Calliphoridae species-specific SNPs and 4 Sarcophagidae species-specific SNPs were selected within the mitochondrial* COI* gene locus. With the combination of these 6 SNPs, it is possible to distinguish between 11 Calliphoridae species, and the combination of 4 SNPs can be used to distinguish 7 Sarcophagidae species (Tables 4 and 5).

### 3.2. Detection of SNP Markers

#### 3.2.1. SNaPshot Template Amplification by Singleplex PCR

Genomic DNA extracted from the 11 Calliphoridae species was amplified using the CA-SNP primer set. The amplification of these DNA fragments was confirmed with a 2% agarose gel. The fragment sizes of the PCR products were approximately 353 bp. The genomic DNA of the 7 Sarcophagidae species was amplified using the SA-SNP primer set. The 151-bp amplifications were performed, and the quality was checked by gel electrophoresis in a 2% agarose gel.

#### 3.2.2. SNaPshot Multiplex Reaction

Eleven primers (6 universal primers to target the Calliphoridae species and 5 other primers to detect low signals in some species) were used to distinguish the Calliphoridae species, and 4 primers were used to distinguish the Sarcophagidae species. Eleven Calliphoridae species could be distinguished by comparing the genotypes from 6 SNP sites. To distinguish 7 Sarcophagidae species, 4 SNP sites were sufficient. The CA-SNaPshot multiplex reaction results for the 11 Calliphoridae fly species were as expected (Supplementary Figures 1-1, 1-2, and 1-3). The SA-SNaPshot multiplex reaction results for the 7 Sarcophagidae fly species were also as expected (Supplementary Figures 2-1 and 2-2).

### 3.3. SNaPshot Assay Validation

#### 3.3.1. Reproducibility Test

The reproducibility of the system was determined by preparing various sample types (larva, pupa, and fly). The 116 Calliphoridae DNA samples from 54 voucher flies, 4 pupae, and 58 larvae were identified based on the combination of 6 SNPs through the CA-SNaPshot multiplex system. The 69 Sarcophagidae DNA samples from 43 voucher flies and 26 larvae were validated based on the combination of 4 SNPs through the SA-SNaPshot multiplex system. All Calliphoridae and Sarcophagidae specimens matched perfectly when compared to the expected SNP combinations (Table 6).

#### 3.3.2. Accuracy Test

When the flies identified by morphology and sequencing methods were applied to this SNaPshot multiplex assay, the results 100% matched. (Table 6). Furthermore, each sample correctly showed the expected combinations of SNP typing as predicted. Thus, 116 Calliphoridae flies and 69 Sarcophagidae flies were correctly identified with the SNaPshot multiplex assay (Table 6). The observed range and standard deviations of peak sizes for each single signal are shown in Tables 4 and 5. These results confirmed the high concordance of the CA and SA SNaPshot multiplex systems.

## 4. Discussion

This SNaPshot multiplex system, based on multiplex single-base primer extension reactions, is very useful in the forensic science field because of its capacity for high precision with a low starting concentration of DNA in a short time frame [25]. Compared with Sanger's nucleotide sequencing, this system is more appropriate for effective typing in forensics. In this study, we focused on the identification of forensically important fly species using the SNaPshot multiplex system. The target interspecific SNPs were selected by comparing the consensus* COI* nucleotide sequences, which include all the intraspecific SNPs collected from the NCBI GenBank database.

As shown in the results, it is remarkable that the combination of 6 SNPs successfully distinguished 11 Calliphoridae species, and the combination of 4 SNPs perfectly distinguished 7 Sarcophagidae species. In addition, the system did not detect any nucleotide combinations that differed from the expected results. Concerning the fragment sizes of these SNPs, the observed peak size was larger than the actual expected peak size, although it remained within 5 nucleotide bases. The size difference between them was predicted based on dye mobility, nucleotide composition, and fragment size in the capillary electrophoresis; the smaller the fragment size is, the greater the impact of the fluorescent dye is [26].

A reproducibility test of the system was performed, which is necessary when using samples from various developmental stages (adult, larva, pupa). Moreover, the SNaPshot multiplex reaction results for 116 Calliphoridae samples and 69 Sarcophagidae samples were computed as expected nucleotide combinations. Therefore, these SNaPshot multiplex systems have perfect reproducibility. The precision of the system was also confirmed. The SNaPshot multiplex reaction results matched 100% with Sanger's sequencing databases for all samples, and the standard deviation of peak positions was between 0.18 and 0.85 at the observed peak size. These results confirmed the high concordance of the CA and SA SNaPshot multiplex method.

The SNaPshot multiplex system is appropriate for the forensic science field; it does not require a high DNA concentration, and it saves time. In addition, it is very convenient, as it does not require a phylogenetic tree. Therefore, this method may be easily used to identify two forensically important families (Calliphoridae and Sarcophagidae) collected in Korea. This study is the first of its kind, and the findings may be used in future technology. We will attempt to increase the number of SNPs in further studies to increase the specificity and sensitivity of identification. Additionally, because this identification system only covers flies collected in Korea, coverage of foreign fly species will be required.

## Figures and Tables

**Table 1:** Nucleotide sequences used for design of SNaPshot primers.

Family	Species	NCBI accession number	Authors of relevant papers	Year published	Coverage on *COI* gene
Calliphoridae	*Lucilia ampullacea*	EU925394, DQ453487, EU418575	Hwang JJ et al., Wells JD et al., Harvey ML et al.	2009, 2006, 2008	Full, 7-end, 1–1167
	*Lucilia caesar*	EU880193–EU880196, KM657111–KM657113	Park SH et al., Schoofs K et al.	2009, 2014	1–1359
	*Triceratopyga calliphoroides*	EU880176–EU880179	Park SH et al.	2009	Full
	*Lucilia illustris*	KM657109–KM657110, EU880197–EU880205	Schoofs K et al., Park SH et al.	2014, 2009	1–1488, full
	*Calliphora lata*	EU880183–EU880187	Park SH et al.	2009	Full
	*Chrysomya megacephala*	JX913788–JX913739, AJ426041	Nelson LA et al., Stevens JR et al.	2012, 2008	Full
	*Chrysomya pinguis*	KM873620, KM244730, AY092759	Park SH et al., Yan J et al., Chen WY et al.	2014, 2014, 2004	Full, 1–1534, full
	*Phormia regina*	KF225240–KF225248	GilArriortua M et al.	2014	1–1274
	*Lucilia sericata*	JX913754–JX913757, EU880208–EU880212	Nelson LA et al., Park SH et al.	2012, 2009	1–1534, full
	*Calliphora vicina*	EU880188–EU880192, JX913760, KF918981	Park SH et al., Nelson LA et al., Sonet G et al.	2009, 2012, 2013	Full, 1–1534, 1–1534
	*Aldrichina grahami*	EU880181, KP872701	Park SH et al., Guo YD et al.	2009, 2015	Full, 1–1534

Sarcophagidae	*Parasarcophaga albiceps*	JX861469–JX861473, KT444443	Kim YH et al., Guo YD et al.	2014, 2015	Full, 1–1534
	*Sarcophaga dux* ^∗^	JX861474–JX861475, EF405937–EF405939	Kim YH et al., Tan SH et al.	2014, 2010	Full, 7-end
	*Sarcophaga haemorrhoidalis* ^∗∗^	KM881633, JX861406–JX861408	Fu X et al., Kim YH et al.	2014	1–1534, full
	*Sarcophaga melanura*	KP091687, JX861418–JX861419	Zhang C et al., Kim YH et al.	2014	1–1534, full
	*Sarcophaga peregrina*	JX861409–JX861412, KF921296	Kim YH et al., Zhong M et al.	2014	Full, 1–1534
	*Sarcophaga similis*	KM287431, JX861476–JX861480	Cai JF et al., Kim YH et al.	2014	1–1534, full
	*Sarcophaga crassipalpis*	KC005711, KJ420597–KJ420599	Ramakodi MP et al., Guo YD et al.	2012, 2014	1–1534, 7-end

^*∗*^ *Sarcophaga dux*: the revised name of *Sarcophaga harpax *in the previous study [7]; ^*∗∗*^*Sarcophaga haemorrhoidalis* (syn): *Sarcophaga africa* (Wiedemann, 1824).

**Table 2:** Primer sequences used for the amplification and SNaPshot multiplex reaction of Calliphoridae and Sarcophagidae *COI* genes.

Family	Name	Sequence	Binding site
Calliphoridae	CA-SNP-F	5′-CAGTCTATTGCCTAAACTTCAG-3′	tRNA-tyrosine
	CA-SNP-R	5′-GTTA**R**TGC**R**GG**R**GGTAAAAGTCA-3^′∗^	301–323 on* COI *
Sarcophagidae	SA-SNP-F	5′-AAGTTTAG**Y**ATC**H**CAACG**W**CAAGT-3^′∗^	1416–1439 on *COI *
	SA-SNP-R	5′-TTAAACCCATTGCACTAATCTGCC-3^′∗^	1543–1566 on *COI *

^*∗*^Degenerated primers were used to detect target SNPs based on IUPAC nucleic acid sequences.

**Table 3:** SNP locations in the mitochondrial *COI *gene and SNaPshot extension primer sequences used in this study. The size in terms of nucleotide bases and the optimal primer concentration in the SNaPshot multiplex are also shown.

SBE system	Location	SBE primer sequence (5′ → 3′)	Primer size (nucleotides)	[Opt.^‡^] (*μ*M)
Calliphoridae	CA90	CTTGATC**N**GGAATA**R**T**H**GGAACTTC^∗^	25	0.80
		CTTGATCCGGTATAAT**Y**GGAACTTC^∗†^	25	0.01
		CTTGATCAGGAATAATTGGTACTTC^†^	25	0.20
	CA72	TTTTTTACTTTATA**Y**TTTATTTTTGGAGCTTGATC^∗^	35	0.04
		TTTTTTACTTTATATTT**Y**ATTTTCGGAGCTTGATC^∗†^	35	0.06
	CA168	TTTTTTTTTTTTTTTTGGAGA**Y**GA**Y**CAAATTTATAATGTAATTGT^∗^	45	0.10
	CA261	TTTTTTTTTTTTTTTTTTTTTTTTTTAATTGATTAGTTCC**W**TTAATACTAGG**R**GC^∗^	55	0.20
		TTTTTTTTTTTTTTTTTTTTTTTTTTAATTGA**Y**TAGTTCCTTTAATGTTAGGAGC^∗†^	55	0.10
		TTTTTTTTTTTTTTTTTTTTTTTTTTAATTGATTAGT**Y**CCTTTAATATTAGGAGC^∗†^	55	0.13
	CA252	TTTTTTTTTTTTTTTTTTTTTTTTTTTTTTTTTGGAGG**R**TTTGGAAATTGA**Y**TAGT**Y**CC**W**TTAAT^∗^	65	0.14
	CA243	TTTTTTTTTTTTTTTTTTTTTTTTTTTTTTTTTTTTTTTTTTTTTTTTTTTTGGAGG**R**TT**Y**GG**W**AATTGA**Y**TAGT^∗^	75	1.80

Sarcophagidae	SA1491	AATTCAGAATAACTATGTTCAGCTGG	26	0.06
	SA1488	TTTTTTTTTTTTTGAATAACTATGTTCAGCTGG**H**GG^∗^	36	0.15
	SA1479	TTTTTTTTTTTTTTTTTTAACTATGTTCAGCTGG**H**GG**D**GT**R**TTTTG^∗^	46	0.30
	SA1485	TTTTTTTTTTTTTTTTTTTTTTTTTTTTTTTTTTTTCTATGTTCAGCTGG**H**GG**D**GT^∗^	56	0.40

^*∗*^Degenerated primers were used to detect target SNPs based on IUPAC nucleic acid sequences; ^†^additional primers for samples with low peak signal sites; ^‡^[Opt.]: optimal concentration of primer.

**Table 4:** Expected nucleotide base, dye color, expected and observed peak size, range of obtained peak sizes, and standard deviation for Calliphoridae species-specific SNPs.

Fly species (Calliphoridae)	Callipho 90		Callipho 72		Callipho 168		Callipho 261		Callipho 252		Callipho 243	
	Color	Nucleotide base	Color	Nucleotide base	Color	Nucleotide base	Color	Nucleotide base	Color	Nucleotide base	Color	Nucleotide base
*Lucilia ampullacea *(Villeneuve, 1922)	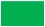	A	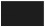	C	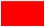	T	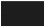	C	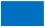	G	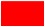	T
*Lucilia caesar *(Linnaeus, 1758)	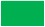	A	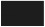	C	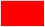	T	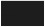	C	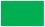	A	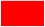	T
*Lucilia illustris *(Meigen, 1826)	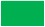	A	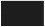	C	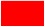	T	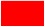	T	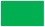	A	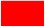	T
*Chrysomya megacephala *(Fabricius, 1794)	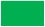	A	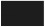	C	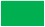	A	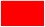	T	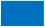	G	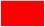	T
*Chrysomya pinguis *(Walker, 1858)	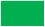	A	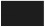	C	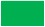	A	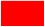	T	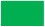	A	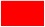	T
*Triceratopyga calliphoroides *(Rohdendorf, 1931)	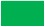	A	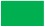	A	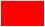	T	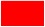	T	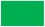	A	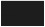	C
*Calliphora lata *(Coquillett, 1898)	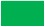	A	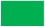	A	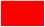	T	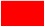	T	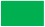	A	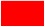	T
*Phormia regina *(Meigen, 1826)	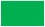	A	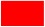	T	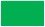	A	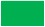	A	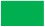	A	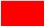	T
*Calliphora vicina *(Robineau-Desvoidy, 1830)	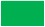	A	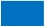	G	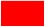	T	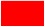	T	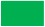	A	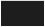	C
*Lucilia sericata *(Meigen, 1826)	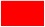	T	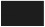	C	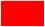	T	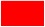	T	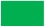	A	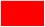	T
*Aldrichina grahami *(Aldrich, 1930)	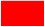	T	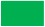	A	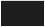	C	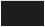	C	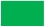	A	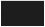	C

Expected peak size	25		35		45		55		65		75	
Observed peak size average	28.73		38.21		48.59		57.79		67.66		78.61	
Observed peak size-range (*n* = 116)	27.96–30.59		37.52–39.37		47.80–49.17		56.93–58.47		66.73–68.32		76.68–79.21	
STD (*n* = 116)	0.55		0.49		0.40		0.35		0.36		0.39	

**Table 5:** Expected nucleotide base, dye color, expected and observed peak size, range of obtained peak sizes, and standard deviation for the Sarcophagidae species-specific SNPs.

Fly species (Sarcophagidae)	Sarco 1491		Sarco 1488		Sarco 1479		Sarco 1485	
	Color	Nucleotide base	Color	Nucleotide base	Color	Nucleotide base	Color	Nucleotide base
*Parasarcophaga albiceps *(Meigen, 1826)	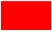	T	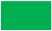	A	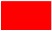	T	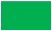	A
*Sarcophaga similis *(Meade, 1876)	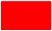	T	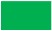	A	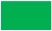	A	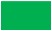	A
*Sarcophaga haemorrhoidalis *(Fallén, 1817)^ ∗∗^	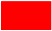	T	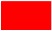	T	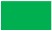	A	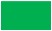	A
*Sarcophaga peregrina *(Robineau-Desvoidy, 1830)	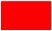	T	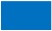	G	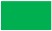	A	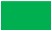	A
*Sarcophaga melanura *(Meigen, 1826)	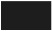	C	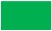	A	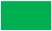	A	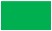	A
*Sarcophaga crassipalpis *(Macquart, 1839)	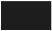	C	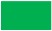	A	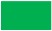	A	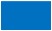	G
*Sarcophaga dux *(Thomson, 1869)^ ∗^	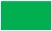	A	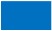	G	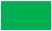	A	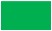	A

Expected peak size	26		36		46		56	
Observed peak size average	30.51		40.76		50.17		59.25	
Observed peak size-range (*n* = 69)	29.02–31.25		39.69–41.53		49.84–50.69		57.57–60.04	
STD (*n* = 69)	0.85		0.63		0.18		0.82	

^*∗*^ *Sarcophaga dux*: the revised name of *Sarcophaga harpax* in the previous study [7]; ^*∗∗*^*Sarcophaga haemorrhoidalis* (syn): *Sarcophaga africa* (Wiedemann, 1824).

**Table 6:** Concordance test between the sequencing and SNaPshot multiplex systems.

Family	Species name	Success rate (%)	Typed/total	Comparing pairs (*n*)	Concordance (%)
Calliphoridae	*Lucilia ampullacea *(Villeneuve, 1922)	100	7/7	7	100
	*Lucilia caesar *(Linnaeus, 1758)	100	11/11	11	100
	*Triceratopyga calliphoroides* (Rohdendorf, 1931)	100	10/10	10	100
	*Lucilia illustris *(Meigen, 1826)	100	10/10	10	100
	*Calliphora lata *(Coquillett, 1898)	100	10/10	10	100
	*Chrysomya megacephala *(Fabricius, 1794)	100	11/11	11	100
	*Chrysomya pinguis *(Walker, 1858)	100	10/10	10	100
	*Phormia regina *(Meigen, 1826)	100	10/10	10	100
	*Lucilia sericata *(Meigen, 1826)	100	11/11	11	100
	*Calliphora vicina *(Robineau-Desvoidy, 1830)	100	10/10	10	100
	*Aldrichina grahami *(Aldrich, 1930)	100	5/5	5	100

Sarcophagidae	*Parasarcophaga albiceps *(Meigen, 1826)	100	8/8	8	100
	*Sarcophaga dux *(Thomson, 1869)	100	10/10	10	100
	*Sarcophaga haemorrhoidalis *(Fallén, 1817)	100	6/6	6	100
	*Sarcophaga melanura *(Meigen, 1826)	100	5/5	5	100
	*Sarcophaga peregrina *(Robineau-Desvoidy, 1830)	100	11/11	11	100
	*Sarcophaga similis *(Meade, 1876)	100	11/11	11	100
	*Sarcophaga crassipalpis *(Macquart, 1839)	100	11/11	11	100

## Supplementary Materials

Supplementary Materials Supplementary Figure  1-1: multiplex system results for Calliphoridae species using Calliphoridae (CA) SNaPshot. Supplementary Figure  1-2: multiplex system results for Calliphoridae species using Calliphoridae (CA) SNaPshot. Supplementary Figure  1-3: multiplex system results for Calliphoridae species using Calliphoridae (CA) SNaPshot. Supplementary Figure  2-1: multiplex system results for Sarcophagidae species using Sarcophagidae (SA) SNaPshot. Supplementary Figure  2-2: multiplex system results for Sarcophagidae species using Sarcophagidae (SA) SNaPshot.
