# Supplementary material for: Identification of Forensically Important Calliphoridae and Sarcophagidae Species Collected in Korea Using SNaPshot Multiplex System Targeting the Cytochrome c Oxidase Subunit I Gene
Source: Biomed Res Int. 2018 Feb 28;2018:2953892. doi: 10.1155/2018/2953892 (PMC5851021; doi:10.1155/2018/2953892)
Supplement: Supplementary Materials — Supplementary Figure 1-1: multiplex system results for Calliphoridae species using Calliphoridae (CA) SNaPshot. Supplementary Figure 1-2: multiplex system results for Calliphoridae species using Calliphoridae (CA) SNaPshot. Supplementary Figure 1-3: multiplex system results for Calliphoridae species using Calliphoridae (CA) SNaPshot. Supplementary Figure 2-1: multiplex system results for Sarcophagidae species using Sarcophagidae (SA) SNaPshot. Supplementary Figure 2-2: multiplex system results for Sarcophagidae species using Sarcophagidae (SA) SNaPshot. [file 2953892.f1.pdf]

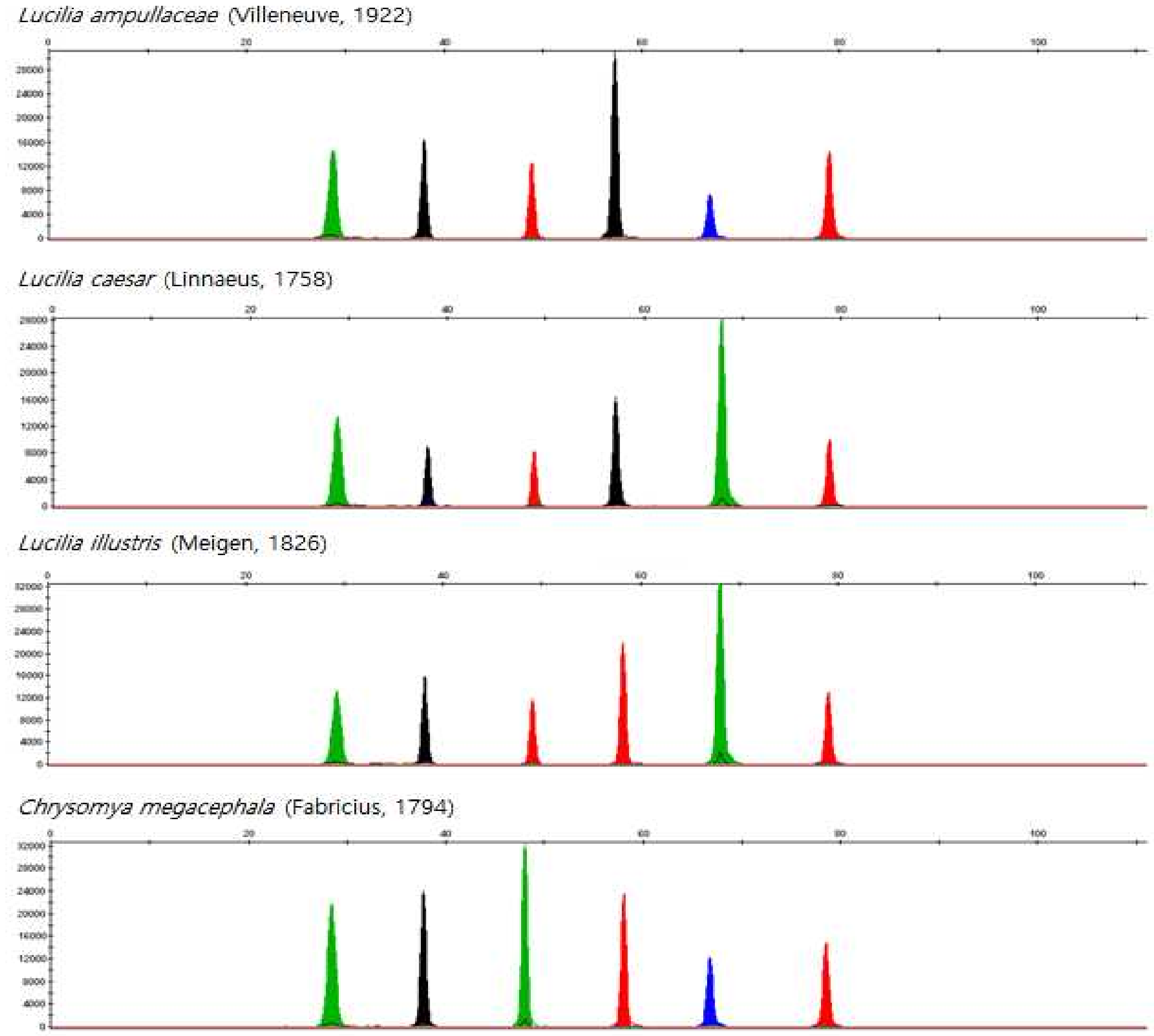

Supplementary Figure 1-1. Multiplex system results for Calliphoridae species: using Calliphoridae (CA)-SNaPshot.

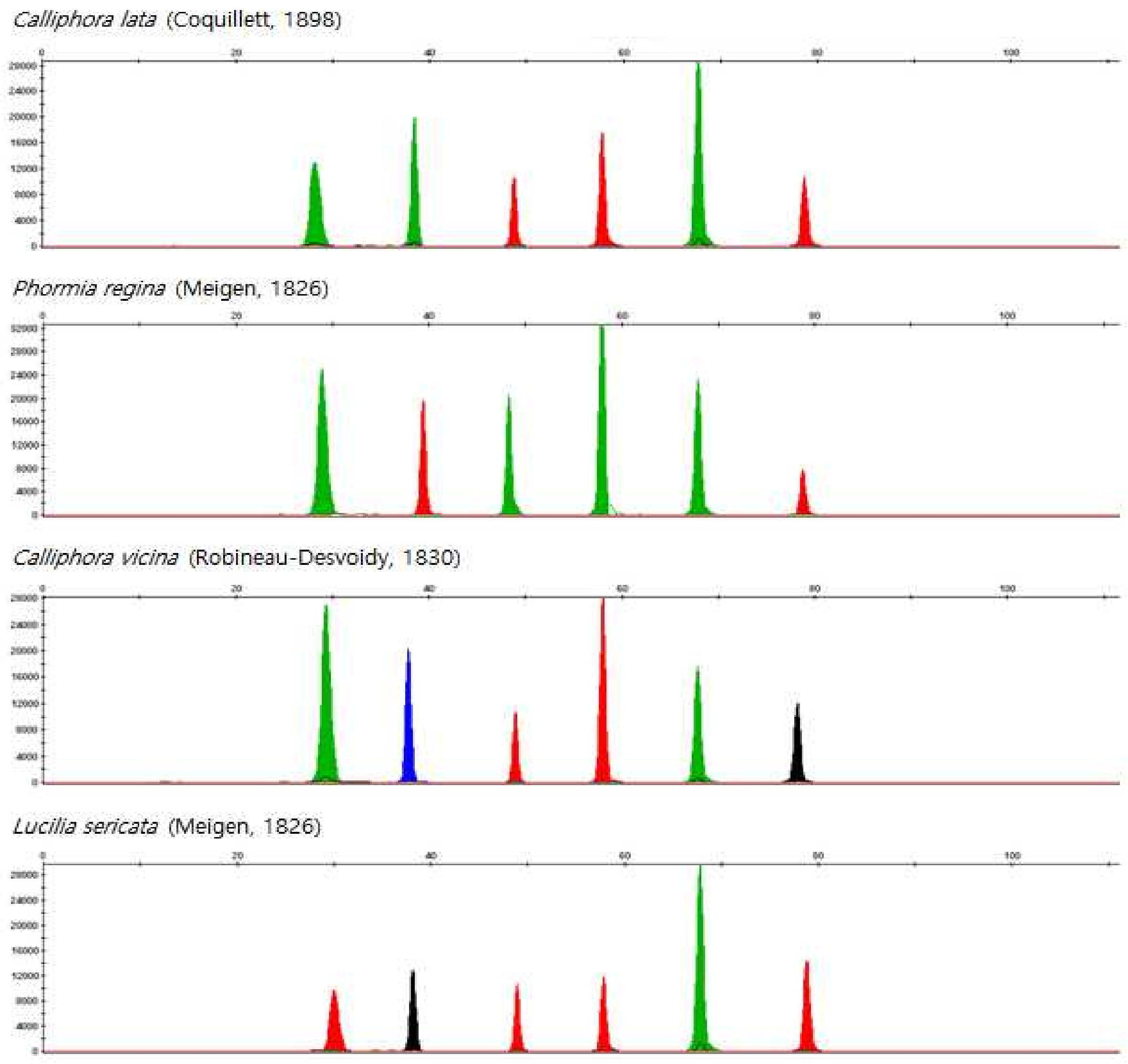

Supplementary Figure 1-2. Multiplex system results for Calliphoridae species: using Calliphoridae (CA)-SNaPshot.

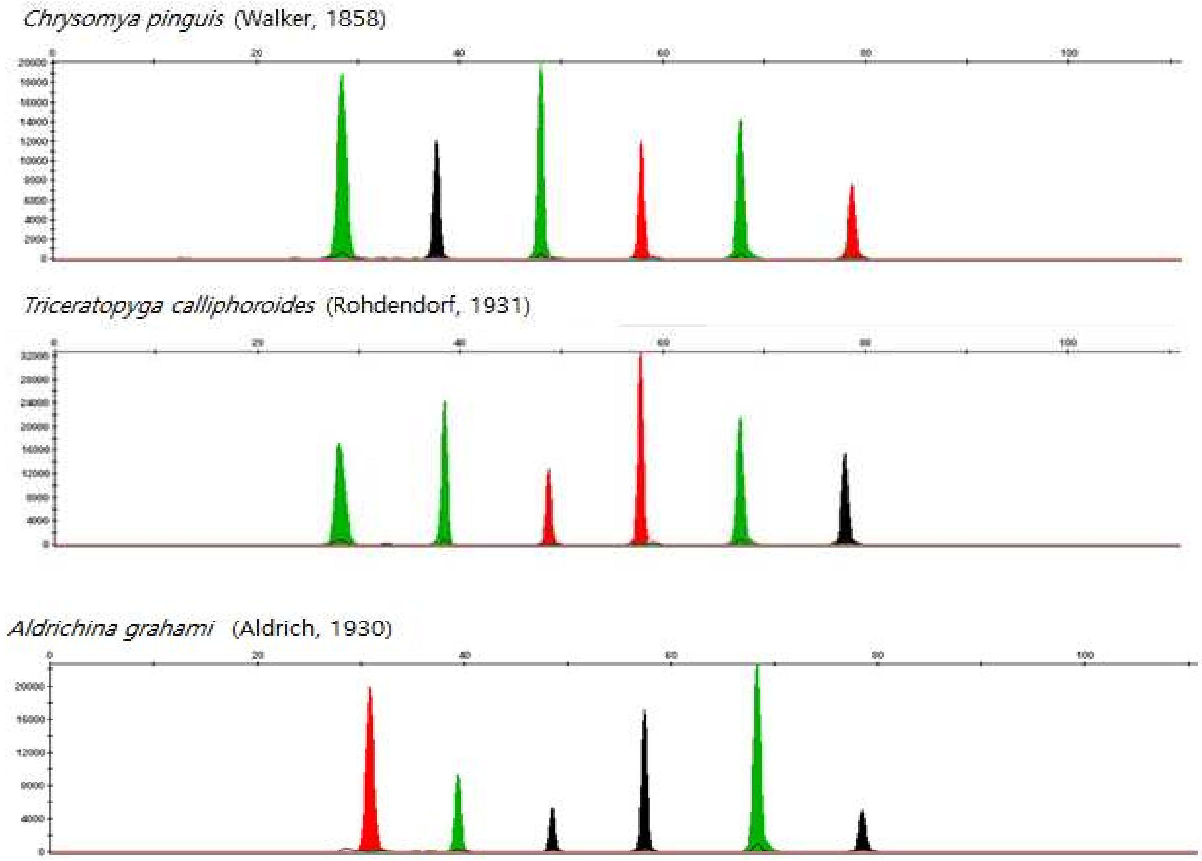

Supplementary Figure 1-3. Multiplex system results for Calliphoridae species: using Calliphoridae (CA)-SNaPshot.

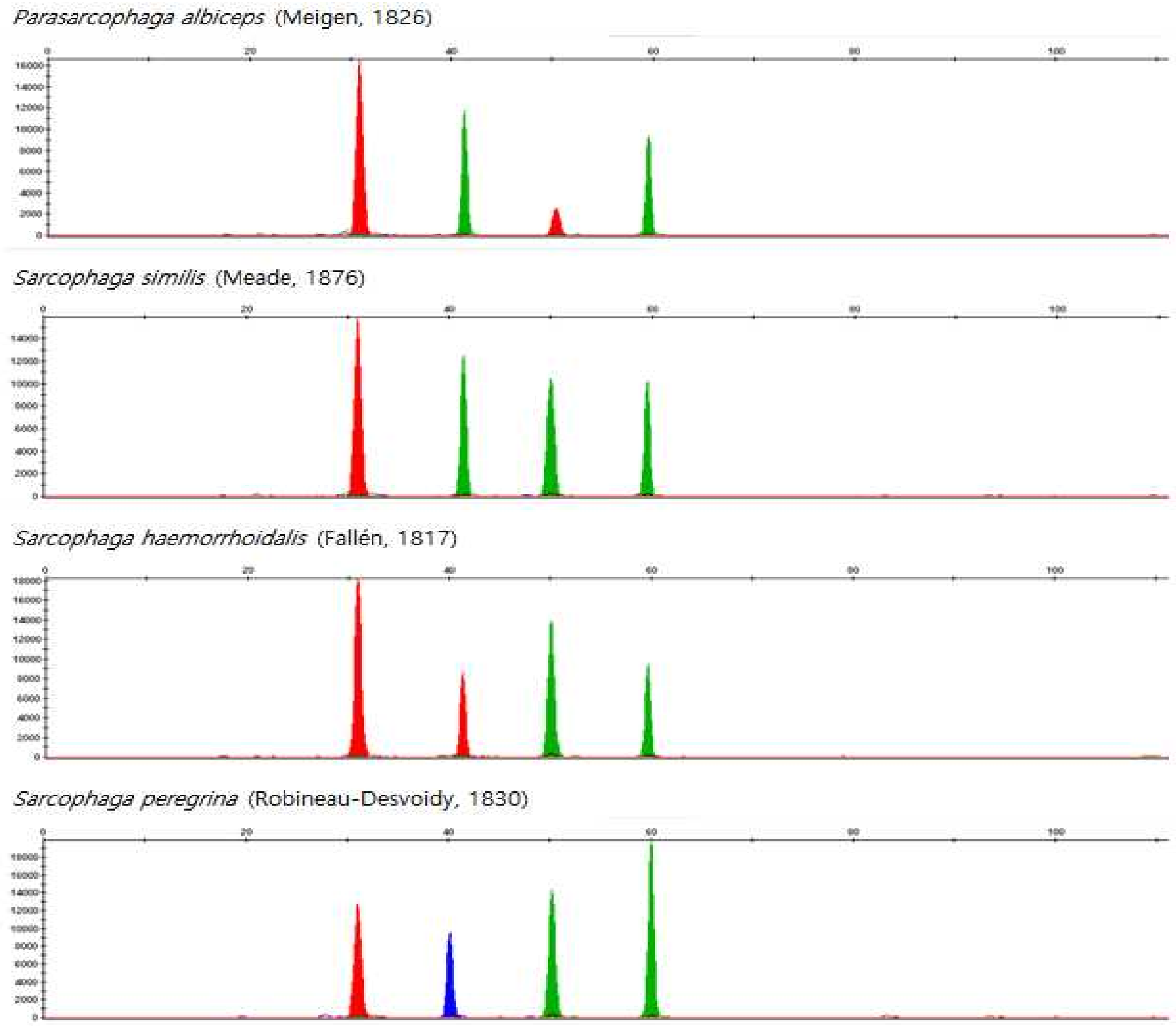

Supplementary Figure 2-1. Multiplex system results for Sarcophagidae species: using Sarcophagidae (SA)-SNaPshot.

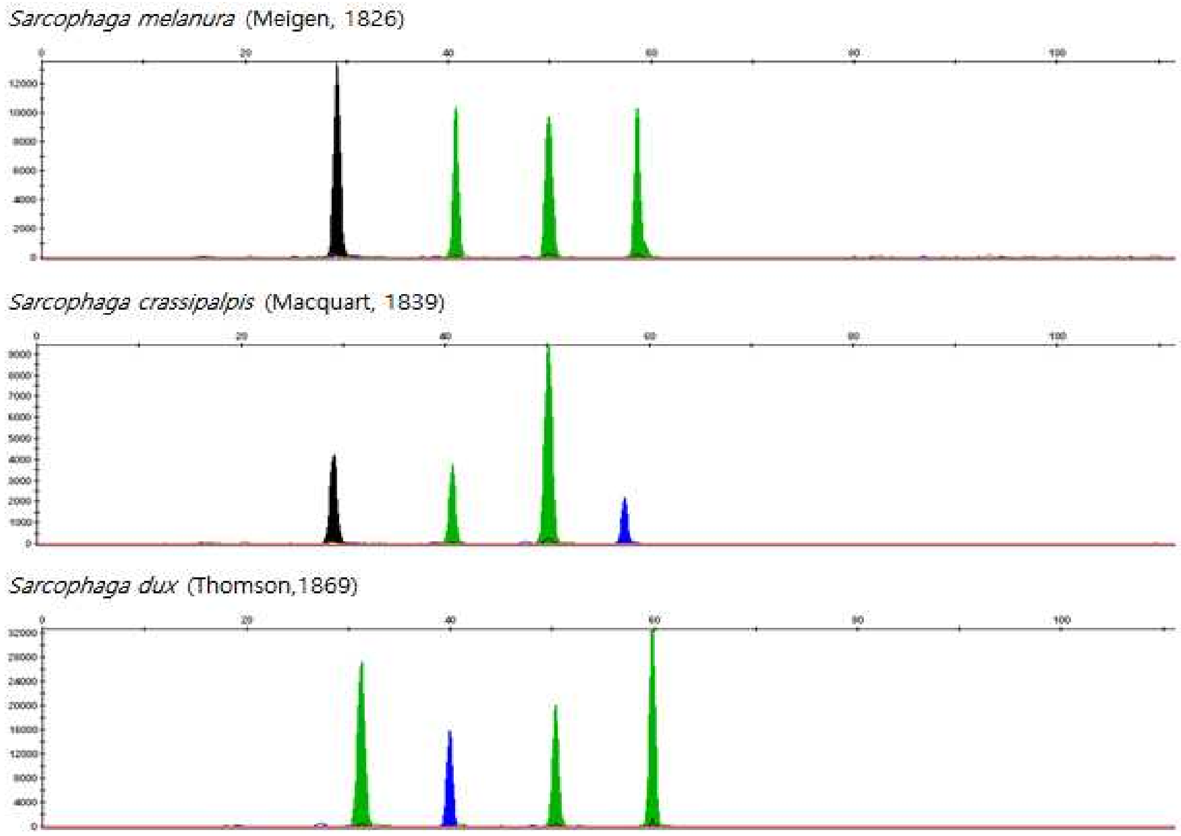

Supplementary Figure 2-2. Multiplex system results for Sarcophagidae species: using Sarcophagidae (SA)-SNaPshot.
